# Single-cell RNA sequencing reveals adrb1 as a sympathetic nerve-regulated immune checkpoint driving T cell exhaustion and impacting immunotherapy in esophageal squamous cell carcinoma

**DOI:** 10.3389/fimmu.2025.1520766

**Published:** 2025-05-08

**Authors:** Qun Li, Shuning Xu, Yulin Ren, Cheng Zhang, Ke Li, Ying Liu

**Affiliations:** Department of Medical Oncology, The Affiliated Cancer Hospital of Zhengzhou University & Henan Cancer Hospital, Zhengzhou, China

**Keywords:** esophageal squamous cell carcinoma, adrenergic receptor, single-cell RNA sequencing, ADRB1 + T cells, prognosis

## Abstract

**Background:**

Esophageal squamous cell carcinoma (ESCC) presents significant health challenges due to its aggressive nature and poor prognosis from late-stage diagnosis. Despite these challenges, emerging therapies like immune checkpoint inhibitors offer hope. β1-adrenergic signaling has been implicated in T cell exhaustion, which weakens the immune response in ESCC. Blocking this pathway could restore T cell function. Recent advances in single-cell RNA sequencing (scRNA-seq) have enabled deeper insights into tumor heterogeneity and the immune landscape, opening the door for personalized treatment strategies that may improve survival and reduce resistance to therapy.

**Methods:**

We combined scRNA-seq with bulk RNA analysis to explore adrenergic receptor signaling in ESCC, focusing on changes before and after neoadjuvant therapy. We identified ADRB1^+^ T cells through data analysis and experimental validation. Copy number variation (CNV) analysis detected malignant cells within scRNA-seq data, while intercellular interaction analysis examined communication between cell populations. Deconvolution of TCGA data revealed key immune populations, which were integrated into a prognostic model based on the adrenergic receptor signaling pathway and differentially expressed genes.

**Results:**

The adrenergic receptor signaling pathway was found in various immune cells, including T cells. scRNA-seq analysis revealed increased ADRB1 expression in T cells after neoadjuvant therapy. Immunofluorescence confirmed colocalization of ADRB1 with T cells, and fluorescence-activated cell sorting (FACS) showed that ADRB1 expression was elevated alongside exhaustion markers, while immune function markers were reduced. CNV analysis highlighted malignant cells in the tumor microenvironment, and intercellular interaction analysis explored ADRB1^+^ T cells’ role in immune support. Deconvolution of TCGA data identified ADRB1^+^ T cells, SPP1^+^ macrophages, and CD44^+^ malignant cells, all of which were prognostically significant. A prognostic model constructed from the intersection of the adrenergic receptor signaling pathway and differentially expressed genes following neoadjuvant therapy showed a significant prognostic effect.

**Conclusions:**

ADRB1 expression increases after neoadjuvant therapy in ESCC and correlates with poor prognosis. Our findings suggest ADRB1 as a potential prognostic biomarker and therapeutic target for post-neoadjuvant immunotherapy.

## 1 Introduction

Esophageal squamous cell carcinoma (ESCC) is a common and aggressive form of esophageal cancer that primarily originates from the squamous epithelial cells lining the upper and middle sections of the esophagus (1, 2). ESCC is one of the two main pathological types of esophageal cancer, the other being esophageal adenocarcinoma, which typically affects the lower esophagus (3, 4). ESCC accounts for roughly 90% of esophageal cancer cases worldwide. The prevalence of ESCC varies by region, with high rates observed in East Asia, parts of Africa, and some areas in South America (5).

The overall prognosis for esophageal cancer remains relatively poor, primarily due to the disease’s tendency to be diagnosed at advanced stages when symptoms become more apparent. For esophageal cancers detected in the early stages, the 5-year survival rate can range from 40% to 50% (5). In cases where the cancer has spread to nearby tissues or regional lymph nodes, the 5-year survival rate decreases to around 20% to 30% (5). Treatment strategies for locally advanced ESCC often includes a combination of surgery, chemotherapy, and radiation. Despite the challenges, ongoing research into new therapies, such as immune checkpoint inhibitors and molecular targeted therapies, continues to offer hope for better management and improved survival rates for esophageal cancer patients in the future (6–8).

T cell exhaustion is a state of functional decline due to prolonged antigen exposure, often seen in the tumor microenvironment (TME) (9, 10). Exhausted T cells exhibit reduced cytokine production, limited proliferative capacity, and high expression of inhibitory receptors like programmed cell death 1 (PD-1) and cytotoxic T-lymphocyte-associated antigen 4 (CTLA-4), all of which impair their ability to attack cancer cells effectively (11–14). Consequently, T cell exhaustion poses a major barrier to successful cancer immunotherapy.

The ablation of β1-adrenergic signaling has gained attention as a potential therapeutic strategy to mitigate T cell exhaustion in cancer (15). β1-adrenergic receptors (β1-ARs), which are primarily involved in cardiovascular function and stress responses, are also expressed on immune cells, including T cells (16–18). These receptors are activated by stress hormones such as norepinephrine, which are often elevated in cancer patients due to the physical and psychological stress associated with the disease (19). Chronic stimulation of β1-ARs in T cells can promote a state of exhaustion by increasing the expression of inhibitory receptors and reducing T cell functionality (20). This adrenergic signaling helps create an immunosuppressive environment within tumors, which can hinder the effectiveness of the body’s immune response and immunotherapies.

The ablation or inhibition of β1-adrenergic signaling, using β1-AR antagonists (commonly known as beta-blockers), may prevent or reverse T cell exhaustion. By blocking β1-ARs, the exhaustion markers on T cells can be reduced, potentially restoring their proliferative and cytotoxic abilities (20, 21). This approach is being explored as an adjunctive strategy to enhance the efficacy of existing cancer therapies, including immune checkpoint inhibitors. Targeting β1-AR signaling may help reshape the TME, fostering a more active and resilient immune response against cancer cells.

Although studies have shown that endogenous expression of *ADRB1* by T cells in melanoma leads to depletion of CD8^+^ T cells, it is still unknown whether endogenous stress response also exists in ESCC to directly regulate the differentiation and function of T cells during immune response.

Single-cell RNA sequencing (scRNA-seq) has transformed cancer research by providing an unprecedented level of detail on individual cell behavior, gene expression, and interactions within tumors. This powerful technology enables the study of cellular heterogeneity, which is crucial for understanding cancer progression, treatment resistance, and therapy response (22, 23). In the context of ESCC and neoadjuvant therapy, scRNA-seq offers valuable insights that can guide personalized treatment approaches and improve patient outcomes (20, 24, 25).

For ESCC, scRNA-seq provides critical insights into the cellular and molecular landscape of the tumor. ESCC is highly heterogeneous, with significant variations in gene expression between different tumor regions and individual cells (26–28). By applying scRNA-seq, researchers can identify specific cell subtypes and states within ESCC tumors that contribute to aggressiveness, metastatic potential, and resistance to therapies (29–31).

Moreover, by profiling immune cells within ESCC tumors, scRNA-seq has shed light on the mechanisms of immune evasion, highlighting interactions between cancer cells and immunosuppressive cells like regulatory T cells (Tregs) and myeloid-derived suppressor cells (MDSCs) (32–34). This knowledge can inform the development of therapies targeting the tumor microenvironment, potentially enhancing immune responses against ESCC.

Neoadjuvant therapy, which includes chemotherapy, radiation, or targeted therapies administered before surgery, aims to reduce tumor size and eliminate micrometastatic disease (35–37). In ESCC, scRNA-seq has revealed changes in gene expression profiles of cancer cells and identified immune cells that are enriched or depleted after therapy, providing clues to the mechanisms behind therapeutic resistance (38).

Furthermore, scRNA-seq can guide the development of biomarkers that predict neoadjuvant therapy responses. By identifying gene expression signatures associated with treatment sensitivity, scRNA-seq helps to personalize therapy regimens based on individual tumor characteristics (25, 39, 40). This precision medicine approach can maximize treatment efficacy, reduce unnecessary side effects, and improve overall survival rates. This study combined single-cell transcriptomics and massive RNA sequencing to investigate the role of ADRB1^+^ T cells after neoadjuvant therapy for ESCC. ADRB1^+^ T cells were found to up-regulate immune checkpoint genes after neoadjuvant therapy, and immunological interactions with other cells occurred. A prognostic model based on adrenergic receptor signaling pathways and cell type characteristics stratifies patients according to risk, demonstrating the potential of ADRB1^+^ T cells as prognostic indicators of ESCC.

## 2 Materials and methods

### 2.1 Datasets source

The single cell transcriptome sequencing data of ESCC before and after neoadjuvant therapy were obtained from China National Center for Bioinformation/Beijing Institute of Genomics, Chinese Academy of Sciences (accession number: OMIX005710) (39). Data are available at https://ngdc.cncb.ac.cn/omix/release/OMIX005710. The bulk RNA-seq data and matching clinicopathological data of 82 ESCC patients were acquired from TCGA (ESCA) (https://portal.gdc.cancer.gov/).

### 2.2 Dimensionality reduction, clustering and differential expression analysis of scRNA-seq data

We used the Scanpy Python package (version 1.9.2) (41) to load the cell-by-gene count matrix and preprocess the data with a modified standard pipeline. First, raw gene counts were normalized for sequencing depth using scanpy.pp.normalize_per_cell, followed by logarithmic transformation with scanpy.pp.log1p. We then identified 10,000 highly variable genes using scanpy.pp.highly_variable_genes, with the flavor parameter set to ‘seurat_v3’ to align with Seurat v3 practices. Dimensionality reduction and batch effect correction were performed with the scVI model (parameters: n_layers = 2, n_latent = 30) from scvi-tools, treating donor information as a batch effect and percent_mito as a numeric covariate. Next, we built the neighborhood graph with scanpy.pp.neighbors (k = 10) and applied the Leiden algorithm (resolution = 1) for cell clustering. Marker genes were detected primarily through a t-test for differentially expressed genes (DEGs) between clusters, using sc.tl.rank_gene_groups (method = ‘t-test_overestim_var’, corr_method = ‘benjamini-hochberg’) with p-values corrected by the Benjamini-Hochberg method, selecting the top 100 genes. DEGs were further filtered to ensure a log2 fold change above one, presence in at least 10% of cells, and a Bayes factor greater than two.

### 2.3 Adrenergic score, HLA score and Exhaust T score

Adrenergic genes, HLA genes and Exhaust T genes were used as inputs to calculate Adrenergic score, HLA score and Exhaust T score through ‘scanpy.tl.score_genes’ model from the package scanpy.

### 2.4 Human esophageal cancer tissue immunofluorescence staining

Tumor tissue was performed on formalin-fixed, paraffin-embedded (FFPE) tumor samples retrospectively collected from 16 patients who had received neoadjuvant chemotherapy at Henan Cancer Hospital. Tissue usage was approved by the Medical Ethics Committee of Henan Cancer Hospital under protocol number 2023-053-001.

In brief, consecutive 4-μm thick sections were deparaffinized, rehydrated, and subjected to antigen retrieval using EDTA solution. Endogenous peroxidase activity was blocked with a 1% hydrogen peroxide solution in methanol at room temperature for 30 minutes. Nonspecific binding was minimized by blocking with 0.3% BSA in TBST, followed by incubation with primary antibodies, including anti-CD8A (1:200, A0663, Abclonal) and anti-ADRB1 (1:100, SE36730, SAB), overnight at 4°C.

After rinsing with PBS, sections were treated with secondary antibodies—Alexa Fluor 488-conjugated anti-mouse IgG (A21202) and Alexa Fluor 555-conjugated anti-mouse IgG (A31570) from Invitrogen—for 60 minutes at room temperature. Following PBS washes, the sections were mounted using Vectashield mounting medium with DAPI.

### 2.5 Immunoblotting

Tumor tissues were lysed in RIPA buffer containing protease inhibitors. Proteins were then separated by SDS-PAGE and transferred onto a PVDF membrane for immunoblot analysis. The membranes were incubated with primary antibodies at a 1:1000 dilution. Protein bands were visualized using the SuperSignal West Pico Kit, following the manufacturer’s instructions (Thermo Fisher Scientific, Pierce).

### 2.6 Flow cytometry

Human esophageal tumor tissues were collected from patients before and after neoadjuvant therapy, minced with razor blades in a cell culture dish, and digested in dissociation buffer (10×) consisting of 40 ml RPMI/DMEM (Gibco) with 1% penicillin–streptomycin (Gibco), 1 mM NaPyr (Gibco), 25 mM HEPES (Lonza), 400 mg collagenase IV (Sigma), 400 mg soybean trypsin inhibitor (Thermo Scientific), 50 mg Dispase II (Sigma), and 20 mg DNase (Sigma) for 30 minutes at 37°C. The samples were then filtered through a 70 μm cell strainer, centrifuged at 420 rcf for 4 minutes at 4°C, and resuspended in RPMI with 10% FBS before being plated for staining.

For flow cytometry staining, cells were incubated with a viability dye (Ghost Dye Red 780, Tonbo) in PBS for 5 minutes at room temperature, followed by incubation with the specified surface antibodies for 30 minutes on ice in PBS supplemented with 1.2% FBS. For intracellular cytokine staining, tumor cells were stimulated with ionomycin (Cell Signaling, final concentration of 1 μg/ml) and PMA (Sigma, final concentration of 50 ng/ml) for 5 hours in the presence of brefeldin A (GolgiPlug, BD Biosciences; 0.5 μl/ml) and monensin (GolgiStop, BD Biosciences; 0.325 μl/ml) for 4 hours at 37°C. Following staining, cells were resuspended in PBS with 2 mM calcium and 2% FBS and incubated with 10 μM noradrenaline or adrenaline for 15 minutes.

### 2.7 Copy number variation analysis

The evaluation of copy number variation (CNV) in scRNA-seq data was performed using the inferCNV R package (version 1.18.0), available from the Broad Institute’s GitHub repository (https://github.com/broadinstitute/inferCNV). This tool facilitates the differentiation of cancerous cells from healthy ones by examining chromosomal regions and gene expression to identify variations in copy number. Cells exhibiting elevated CNV scores were classified as malignant.

### 2.8 Cell communication

The CellChat R package (version 1.5.0) (42) was employed to quantitatively infer and analyze cellular interactions derived from scRNA-seq data. To examine changes in intercellular communication strength, the “netVisual_diffInteraction” function was applied, while the “identifyCommunicationPatterns” function was used to assess the number of distinct communication patterns. Various visualization methods, including scatter plots and heatmaps, were implemented to visually analyze the signals exchanged by each cell.

### 2.9 Running deconvolution on ESCC samples using CIBERSORT

Bulk RNA-seq deconvolution and cell type estimation was performed using CIBERSORTx and the MQC genes signature matrix (43). MQC genes signature matrix comprises 14 genes, derived from aqueous humor outflow pathways in eyes of human scRNA-seq data, to distinguish 15 human cell types.

### 2.10 Single-sample gene set enrichment analysis

The ssGSEA algorithm was based on MQC genes. We employed the ssGSEA algorithm via GSVA (44) packages (version: 1.40.1) to comprehensively assess the MQC characteristics of every sample included in the study.

### 2.11 Independent prognostic evaluation of the risk score and the construction of nomogram

Patient data was sourced from the TCGA-ESCA (ESCC) dataset, with variables and risk scores assessed through univariate and multivariate Cox regression analyses. A nomogram was constructed using the rms package (version: 6.2-0) to visualize independent variables for potential clinical application in predicting the prognosis within the training cohort. The nomogram’s performance was evaluated with both the Decision Curve Analysis (DCA) and calibration curves.

### 2.12 Statistical analysis

All statistical analyses were performed using R software (version 4.3.0) and R studio (version 1.4.1717). Using the Wilcoxon test to compare *ADRB1* gene expression levels in patients before and after neoadjuvant therapy, tumor stemness scores of malignant cells before and after neoadjuvant therapy, and ADRB1^+^ T cell scores across patients with different clinical characteristics in the TCGA cohort. A two-tailed P value <0.05 was considered statistically significant.

## 3 Results

### 3.1 Overview of β1-ARs in scRNA-seq data of ESCC

**Figure 1:**
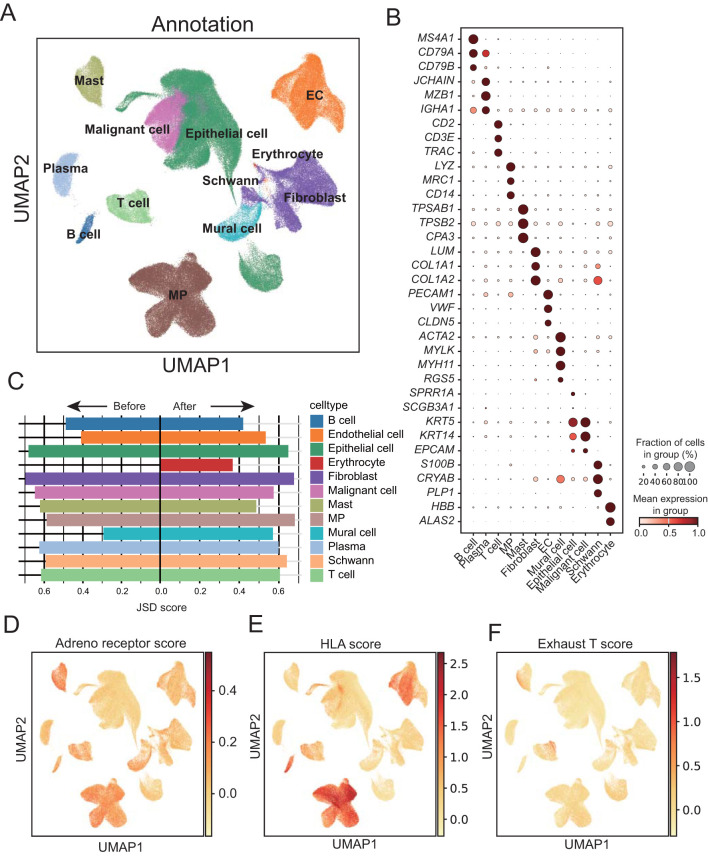
Single-cell landscape of adrenergic receptor in ESCC before and after neoadjuvant therapy. **(A)** Uniform manifold approximation and projection (UMAP) visualization of annotated cells in cell atlas of ESCC tumors. EC, Endothelial cells; MP, mononuclear phagocytes. **(B)** Dot plot showing marker genes for ESCC cell populations. The dot size represents the proportion of cells expressing a gene. Color denotes the scaled expression level. **(C)** Bar chart showing the Jensen–Shannon divergence scores of each cell population. **(D-F)** UMAP plots showing expression of adrenal receptor pathway-related genes, T-cell exhaust-related genes and HLA-related genes enriched in ESCC cancer cells, respectively.

β1-ARs in immune cells have been shown to interfere with their immune function through endogenous stress responses. Therefore, to explore the expression of β1-ARs related genes before and after ESCC neoadjuvant chemotherapy, we analyzed single cells from 22 baseline and 24 post-neoadjuvant chemo-immunotherapy samples of stage II/III ESCC patients (38). After batch removal and rigorous data filtering, we obtained 238,209 cells, including 12 major cell types (**Figure 1A**). These major cell types were annotated based on canonical cell-type marker genes reported in prior studies (**Figure 1B**). The 12 cell types include immune cells (T cells, mast cells, B cells, plasma cells, and mononuclear phagocytes), stromal cells (endothelial cells, mural cells and fibroblasts), epithelial cells (malignant cells and normal epithelial cells), Schwann cells and erythrocytes. Through the analysis of the sample source and tissue distribution of single cells, it was found that the distribution of cells is relatively uniform among patients (**Supplementary Figures S1A, B**). Subsequently, we analyzed the distribution of patients’ treatment status in each cell type, and found that immune cells were significantly elevated in patients after treatment (**Supplementary Figures S1C, D**).

By calculating the degree of cell heterogeneity of each type of cell population before and after neoadjuvant therapy, we calculated the Jensen-Shannon divergence (JSD) score dispersion of the cell population. Through comparison, we found that the heterogeneity of immune cells such as B cells, plasma cells, T cells and mast cells decreased significantly after treatment, while stromal cells such as endothelial cells, Schwann cells and mural cells increased significantly (**Figure 1C**; **Supplementary Table 1**). In order to detect the immune assistance and cell depletion status of different cell types in ESCC, we calculated adrenal receptor pathway-related genes, T-cell exhaust-related genes and HLA-related gene score, respectively, and found that adrenal receptor pathway-related genes were expressed in a variety of immune cells (**Figure 1D**). HLA-related genes were mainly highly expressed in mononuclear phagocytes and B cells (**Figure 1E**). T-cell exhaust-related genes were significantly expressed in some T cells (**Figure 1F**), indicating the presence of a population of exhausted T cells within the T cell population. Furthermore, the cells with high exhausted T score also showed high scores of genes related to the adrenergic receptor signaling pathway.

### 3.2 Microenvironment of ESCC after neoadjuvant therapy

**Figure 2:**
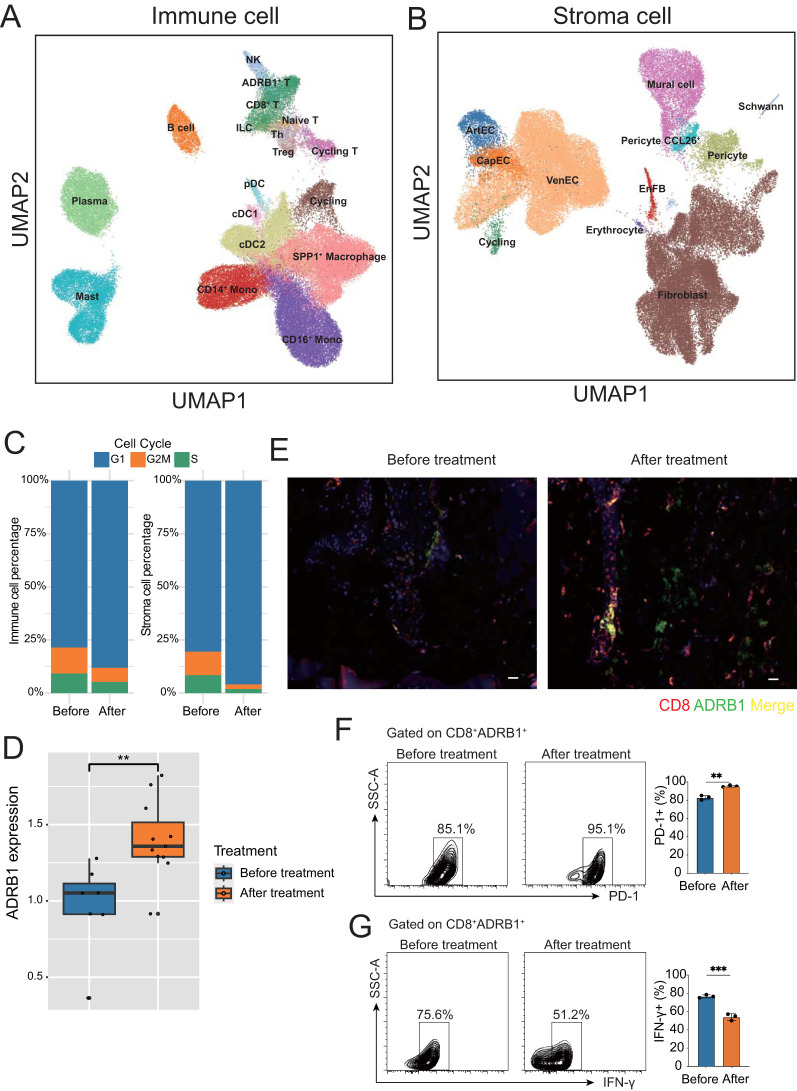
Microenvironment of ESCC before and after neoadjuvant therapy. **(A)** UMAP plot of immune cell subtypes annotation in ESCC tumors. **(B)** UMAP plot of stroma cell subtypes annotation in ESCC tumors. **(C)** Bar chart showing the percentage of immune and stromal cells in different cell cycle stages before and after neoadjuvant therapy. **(D)** Box plot showing the upregulation of ADRB1 expression after neoadjuvant therapy. **(E)** Immunofluorescence staining shows colocalization of ADRB1 with CD8, and a significant upregulation in expression following neoadjuvant therapy, CD8 (red), ADRB1 (green) (scale bar: 25 µm). **(F, G)** Gating strategy for FACS-based human T cell sorting. **P < 0.01, ***P < 0.001.

To further investigate the microenvironment of ESCC after treatment, we separated and finely annotated major cell populations, including immune cells and stroma cells (**Figures 2A, B**; **Supplementary Figures S2A, B**). To detect the degree of dispersion between cells after treatment, we calculated JSD scores for different cell types. The results showed that most cell types, including ADRB1^+^ T cells, had increased cell dispersion after treatment, indicating that intracellular information entropy increased and cell differentiation increased, while B cell and Naïve T cell had decreased divergence, indicating that these cells tended to be homogeneous after treatment (**Supplementary Figure S2C**). In addition, the JSD scores of all stromal cells increased after treatment, indicating that different stromal cells became unhomogeneous after treatment (**Supplementary Figure S2D**). Subsequently, we examined cell proliferation after treatment, and we found that the proliferation capacity of immune cells and stromal cells was generally decreased after treatment (**Figure 2C**; **Supplementary Table 2**).

A previous article reported that ADRB1 is a novel immune checkpoint in colorectal cancer (20). Interestingly, we found a group of T cells specifically expressing ADRB1 in T cells, suggesting that this very exciting group of exhaust-related T cells is also present in ESCC. In addition, ADRB1 expression in T cells were upregulated after treatment (**Figure 2D**), suggesting that neoadjuvant therapy promotes exposure to this immune checkpoint in ESCC. Immunofluorescence confirmed that ADRB1 was expressed in CD8^+^ T cells and that its expression increased after neoadjuvant therapy (**Figure 2E**). Through fluorescence-activated cell sorting (FACS), we found that after neoadjuvant therapy, *ADRB1* and T cell exhaustion-related gene *PD1* were up-regulated, while T cell function related gene *IFN-Y* was down-regulated. These results indicate the presence of a subset of exhausted T cells characterized by the expression of adrenergic receptor ADRB1 in ESCC (**Figures 2F, G**). After neoadjuvant therapy, these cells express more adrenergic receptor-related genes, show an increase in cell numbers, and exhibit a decline in cell function.

### 3.3 The identification of malignant cells

**Figure 3:**
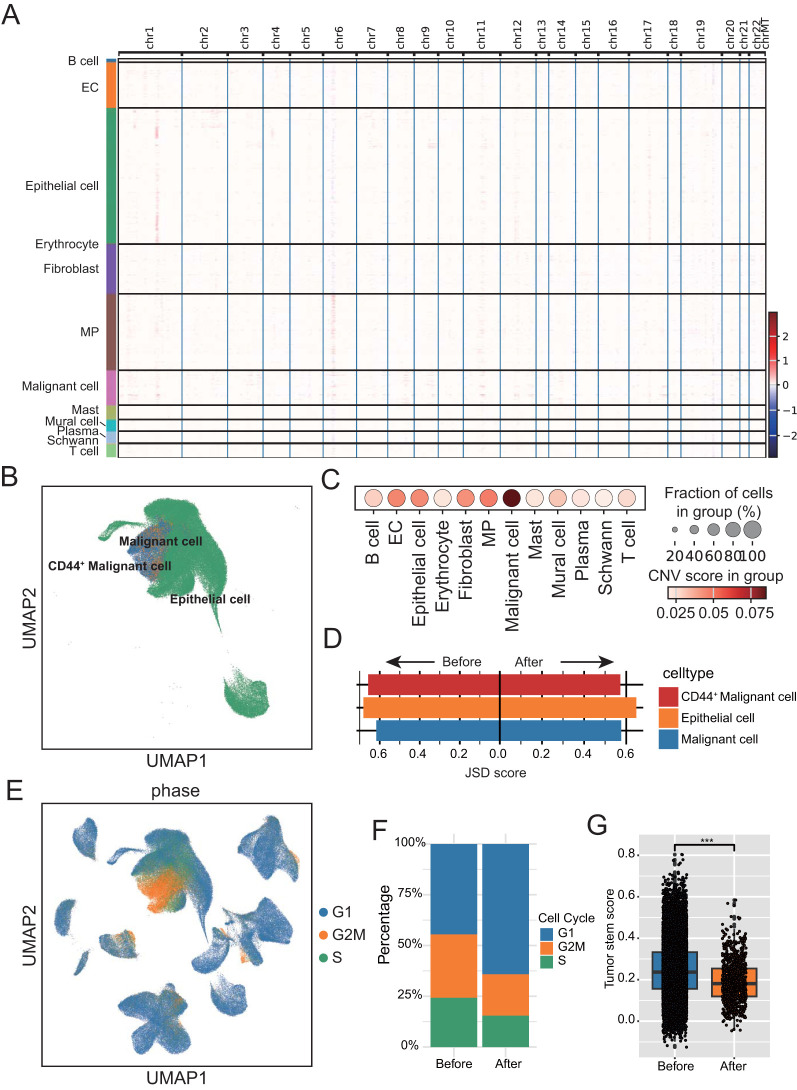
Identification and analysis of malignant cells. **(A)** Heatmap displaying large-scale segmental copy number variations across the genome for each cell population. **(B)** UMAP plot of parenchymal cell subtypes annotation in ESCC tumors. **(C)** Dot plot showing copy number scores for each stromal cell subgroup. The dot size represents the proportion of cells with copy number variation (CNV) score. **(D)** Bar chart showing the Jensen–Shannon divergence scores of each Epithelial cell subtypes. **(E)** UMAP plot showing cell cycle distribution of epithelial and malignant cells. Color denotes the level of the CNV score. **(F)** Bar chart showing the percentage of parenchymal cell in different cell cycle stages before and after neoadjuvant therapy. **(G)** Box-and-whisker plot with points showing a significant decrease in tumor cell stemness following neoadjuvant therapy. ***P < 0.001.

All epithelial cells are divided into classical epithelial cells and unknown cells based on canonical marker genes. inferCNV was then used to detect large-scale chromosomal copy number changes in all epithelial cells using immune cells and stromal cells as reference normal cells. The resulting heatmap illustrated the relative expression intensities across each chromosome, this unknown population expressing showed significant gains or deletions of large segments of chromosomes relative to other normal cells (**Figure 3A**). All epithelial cells were further isolated for re-clustering analysis, and stem cell like CD44^+^ malignant cell was found (**Figure 3B**; **Supplementary Table 2**). Calculating the copy number scores for epithelial cells also revealed that malignant cells had the highest scores (**Figure 3C**). JS divergence analysis showed that both epithelial cells and malignant cells showed a decrease of JSD score after treatment, indicating that the heterogeneity of both epithelial cells and malignant cells was reduced after neoadjuvant therapy (**Figure 3D**). By calculating the proliferation of cells, we found that the vast majority of malignant cells were in the G2M stage (**Figure 3E**). In addition, after treatment, the proliferation of malignant cells showed a significant decline (**Figure 3F**; **Supplementary Table 2**), and the tumor stemness score also showed a significant decline after neoadjuvant therapy (**Figure 3G**, **Supplementary Table 2**).

### 3.4 CellChat analysis among cell subtypes

**Figure 4:**
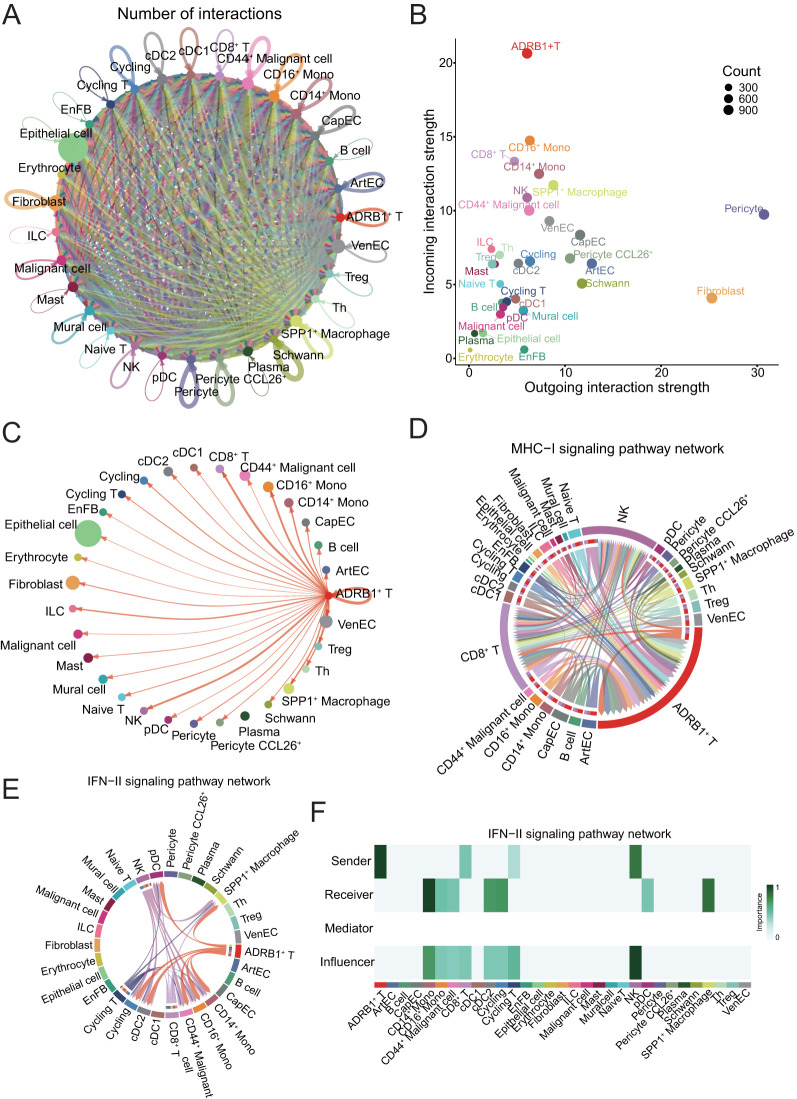
Presentation of CellChat results. **(A)** Circle plot showing the number of interactions among all cells in ESCC. **(B)** Scatter plot showing the communication network analysis between all cells and ADRB1^+^ T subpopulation, the color of the dots indicates different cells, and the size of the dots indicates the number of cells. **(C)** Screening of circle plots depicting cellular interactions, with ADRB1^+^ T cells as the source and tumor cells as the target; line width represents the number of interactions. **(D)** Circle plot showing the strength of cellular interactions between ADRB1^+^ T cell and other tumor cells on MHC-I signaling pathway. **(E)** Circle plot showing the strength of cellular interactions between ADRB1^+^ T cell and other tumor cells on IFN-II signaling pathway. **(F)** Heatmap displaying the interaction relationships between cell populations in the IFN-II signaling pathway.

To deepen our understanding of the communication network between cell types, unravel the complexity of intercellular signaling, and explore the functional and regulatory roles of key cell subpopulations and signaling pathways in both physiological and disease contexts, we employed CellChat for analyzing and visualizing intercellular communication. Firstly, we constructed a communication network among all subtype cells. To assess the level of cellular communication, we evaluated the number of intercellular connections, as indicated by the thickness of the connecting lines (**Figure 4A**). To detect the contribution of cell subtypes to the input and output of intercellular communication, a dot plot was used to show the input and output signal strength of contributions from all cell subclasses (**Figure 4B**).

The results showed that ADRB1^+^ T cells, as incoming cells, were the most strongly regulated by other cells. Subsequently, the interaction between ADRB1^+^ T cells and all other cell subtypes was examined, and the results showed that this group of T cells expressing immune checkpoints had significant interaction with stem cell-like CD44^+^ Malignant cell (**Figure 4C**), indicating that this group of T cells had the effect of directly killing malignant cells, especially stem cell-like malignant cells. The role of ADRB1^+^ T cells in the pathway associated with antigen presentation and cell killing was then examined. The results showed that ADRB1^+^ T cells mainly interact with NK and CD8^+^ T cells on the MHC-I signaling pathway and play an important role in antigen presentation (**Figure 4D**). At the same time, ADRB1^+^ T cells also play an important role as a ligand cell in the IFN-II signaling pathway, and its receptor cells include SPP1^+^ Macrophage (**Figures 4E, F**). Subsequent analysis of CD44^+^ Malignant cell interaction showed that this population had intercellular communication with most cells, among which ADRB1^+^ T cells communicated the most (**Supplementary Figure S3A**). At the same time, tumor-associated SPP1^+^ Macrophage interact with most cells on the SPP1 signaling pathway (**Supplementary Figures S3B, C**).

### 3.5 Single-cell data were deconvolved to bulk TCGA cohort

**Figure 5:**
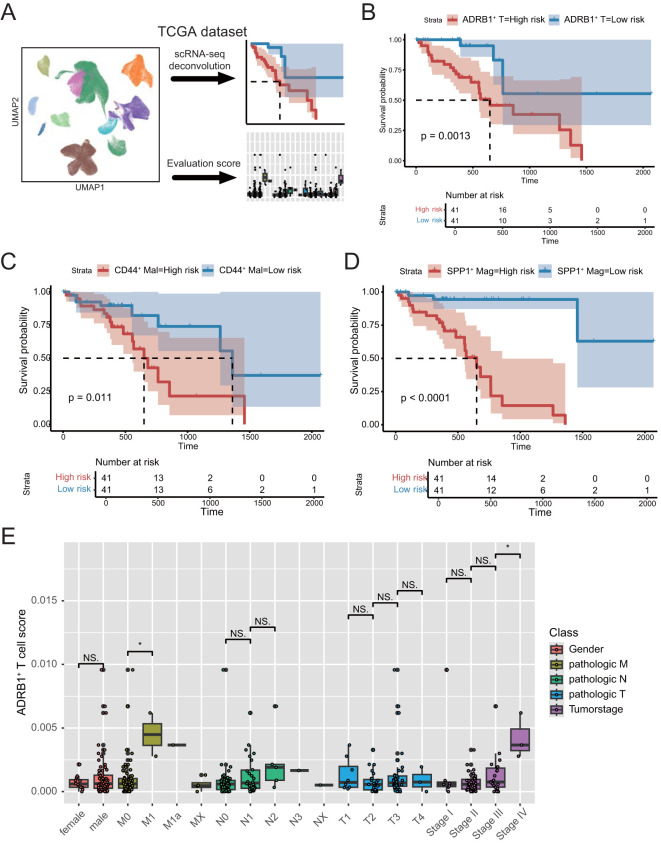
Deconvolution analysis of the TCGA dataset using scRNA-seq data from ESCC. **(A)** Schematic diagram of the deconvolution analysis strategy. **(B)** Kaplan–Meier analyses demonstrate the prognostic significance of the ADRB1^+^ T cell signature in TCGA cohort. **(C)** Kaplan–Meier analyses demonstrate the prognostic significance of the CD44^+^ Malignant cell signature in TCGA cohort. **(D)** Kaplan–Meier analyses demonstrate the prognostic significance of the SPP1^+^ Macrophage signature in TCGA cohort. **(E)** Box plots present differential risk score of multiple clinical character in TCGA cohort. *P < 0.05; NS, not significant.

To assess the impact of these interacting cells on patient prognosis, as well as the prognostic role of the adrenergic receptor pathway in ESCC, we used the expression matrix of ADRB1^+^ T cells, CD44^+^ Malignant cell and SPP1^+^ Macrophage in scRNA-seq data as a feature matrix deconvolution into the bulk RNA-seq expression matrix of ESCC patients in TCGA data, respectively (**Figure 5A**; **Supplementary Table 3**). The median was used to group the deconvolution scores of different cell types, and the results showed that the expression of all three groups of cells could be a significant prognostic indicator for patients with ESCC (**Figures 5B–D**). Subsequently, by analyzing the differences in the ADRB1^+^ T cells deconvolution scores across various clinical characteristics of the patients, the results revealed significant differences in the risk scores across multiple clinical features (**Figure 5E**). This indicates that ADRB1^+^ T cells not only have prognostic value but also show higher risk scores in patients at more advanced stages.

### 3.6 *ADRB1* risk signature was associated with prognosis

**Figure 6:**
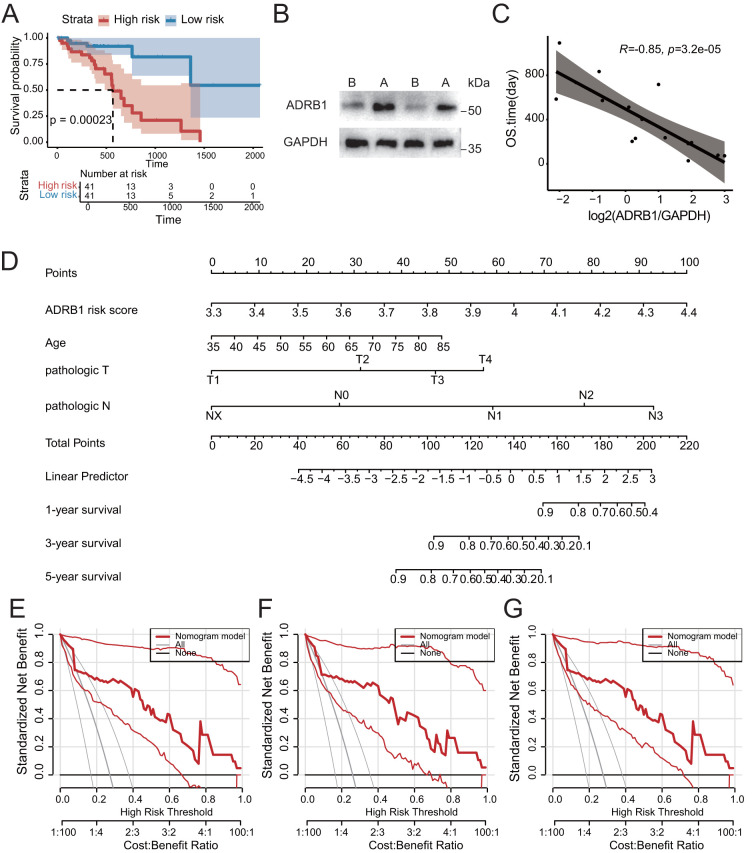
Construction of prognostic model of related features of adrenergic receptor signaling pathway. **(A)** Kaplan-Meier analysis confirmed the prognostic significance of the adrenergic receptor signaling pathway-related features in the TCGA cohort. **(B)** The increase in ADRB1 expression was experimentally validated by Western blotting (ADRB1 protein 51kDa, GAPDH protein ~35kDa). **(C)** Scatter plot showing a significant negative correlation between ADRB1 expression levels and patient survival time following neoadjuvant therapy. **(D)** The nomogram prediction model for ESCC patients’ overall survival. **(E-G)** 1-year, 3-year and 5-year survival benefit in the TCGA cohort.

In order to examine the role of ADRB1^+^ T cells in the clinical prognosis of patients before and after neoadjuvant therapy, we intersected the genes up-regulated by ADRB1^+^ T cells after treatment with the genes of the adrenergic receptor signaling pathway, and then calculated the risk scores of ESCC patients in the TCGA data using ssGSEA (**Supplementary Table 4**). Patients were divided into high-risk and low-risk groups using the median risk score as a threshold, and the Kaplan-Meier survival curve showed significant differences in survival among different groups (**Figure 6A**). On the basis of the results of univariate and multivariate Cox regression analysis, the risk signature was identified to be an independent prognostic factor for overall survival (OS) (P < 0.05) (**Supplementary Figures S4A, B**). Using Western blot to detect ADRB1 expression in tumor tissues of patients after neoadjuvant therapy, the results showed that ADRB1 expression in tumor tissues significantly increased following the therapy (**Figure 6B**). Additionally, a correlation analysis between OS and ADRB1 expression found that ADRB1 expression was significantly negatively correlated with OS (**Figure 6C**; **Supplementary Table 5**). On the basis of four risk factors, a nomogram was constructed to predict prognosis in patients of training cohort (**Figure 6D**). The nomogram showed that the *ADRB1* risk model contributed the most to prognosis. The 1, 3, and 5-year survival calibration curves showed a good consistency between logistic calibration outcomes and predicted outcomes, which demonstrated a good calibration of the diagnostic nomogram (**Supplementary Figures S4C-E**). In addition, the 1, 3, and 5-year survival DCA curves demonstrated a preferable positive net benefit, which suggested its strong clinical utility (**Figures 6E–G**).

## 4 Discussion

ESCC remains a major clinical challenge, particularly given the uncertainty in long-term survival outcomes despite the use of neoadjuvant immune chemotherapy. Although clinical trials such as ESCORT-NEO/NCCES01 indicate that combination immunotherapy (e.g., camrelizumab) can improve pathological complete response (pCR) rates in patients with locally advanced ESCC, the long-term survival benefits remain inconclusive (45). Our study suggests that elevated expression of ADRB1 after neoadjuvant immune chemotherapy correlates with poorer prognosis in ESCC patients, indicating that ADRB1 could serve as an important biomarker for assessing the efficacy of this treatment strategy.

Zhang et al. found that certain genes can regulate the activity of CD8^+^T cells, thereby affecting the anti-tumor immunity of patients (46). The role of ADRB1 in T-cell exhaustion has also been well established in various tumor types. The study demonstrated that the sympathetic nervous system induces functional exhaustion in CD8^+^ T cells via the ADRB1 signaling pathway, thus diminishing the anti-tumor immune response (20). Furthermore, ADRB1 signaling contributes to T-cell exhaustion in the context of chronic infections and tumors, facilitating immune escape (47). These studies provide compelling evidence that ADRB1 is not only a critical mediator of immune suppression within the tumor microenvironment but also a potential therapeutic target to enhance the efficacy of immune treatments.

The molecular mechanisms responsible for the upregulation of ADRB1 expression following neoadjuvant treatment remain to be fully elucidated. Tsai et al. (2020) suggested that chemotherapy and immunotherapy might induce upregulation of ADRB1 on T cells, enhancing their ability to adapt to the tumor microenvironment (48). This could be attributed to the persistent stimulation of T cells by tumor-associated inflammatory cytokines, such as IFN-γ and IL-6, which promote the proliferation of ADRB1^+^ T cells and drive them into an exhausted state (49).

In the tumor microenvironment, cells are usually highly heterogeneous, especially malignant cells. By identifying cell subtypes and analyzing cell interactions, more accurate results are often obtained than by analyzing a wide range of cell types (50–52).

Our study highlights the critical interaction between ADRB1^+^ T cells, SPP1^+^ macrophages, and CD44^+^ malignant cells, providing new insights into the immune microenvironment of ESCC. SPP1^+^ macrophages have been implicated in promoting tumor progression and immune escape (53). Moreover, CD44^+^ malignant cells are widely recognized as crucial factors in maintaining cancer stemness and immune suppression (54). Our findings further suggest that ADRB1^+^ T cells may interact with CD8^+^ T cells and NK cells via the MHC-I signaling pathway, while also forming a complex immune-suppressive network with SPP1^+^ macrophages through the IFN-II signaling pathway. This network could contribute to the development of immune therapy resistance.

The clinical targets identified through data analysis are ultimately to be implemented into clinical applications, which is of great significance for improving the accuracy of prognosis and guiding treatment decisions (51, 52). ADRB1 as a potential therapeutic target has been validated in multiple studies. For example, the study demonstrated that the use of ADRB1 inhibitors, such as atenolol, can reverse T-cell exhaustion and enhance the anti-tumor effects of PD-1 inhibitors (47). In addition, Qiao et al. showed that β-adrenergic receptor antagonists can improve immune therapy responses in various cancer models (55). Therefore, for ESCC patients with elevated ADRB1 expression after neoadjuvant treatment, combining ADRB1 inhibitors with existing immune therapies could be a promising strategy to improve long-term treatment outcomes.

Our study highlights the critical role of ADRB1 in the ESCC tumor microenvironment, particularly in regulating T-cell exhaustion, tumor-immune cell interactions, and immune therapy resistance. Elevated ADRB1 expression could serve as an important marker of poor prognosis following neoadjuvant immune chemotherapy, and ADRB1 inhibition may offer a novel therapeutic approach to enhance the efficacy of immune treatments in ESCC. Future research should further explore the clinical feasibility of ADRB1-targeted therapies in ESCC and investigate their potential synergy with other immune treatment strategies.

## Data Availability

The single cell transcriptome sequencing data presented in the study are deposited in China National Center for Bioinformation/Beijing Institute of Genomics, accession number OMIX005710 (https://ngdc.cncb.ac.cn/omix/release/OMIX005710). The bulk RNA-seq data and matching clinicopathological data of ESCC patients were acquired from TCGA (ESCA)(https://portal.gdc.cancer.gov/).
