# Supplementary material for: Single-cell RNA sequencing reveals adrb1 as a sympathetic nerve-regulated immune checkpoint driving T cell exhaustion and impacting immunotherapy in esophageal squamous cell carcinoma
Source: Front Immunol. 2025 May 8;16:1520766. doi: 10.3389/fimmu.2025.1520766 (PMC12095256; doi:10.3389/fimmu.2025.1520766)
Supplement: Supplementary file 1 [file DataSheet1.pdf]

# *Supplementary Material*

## 1 Supplementary Figures

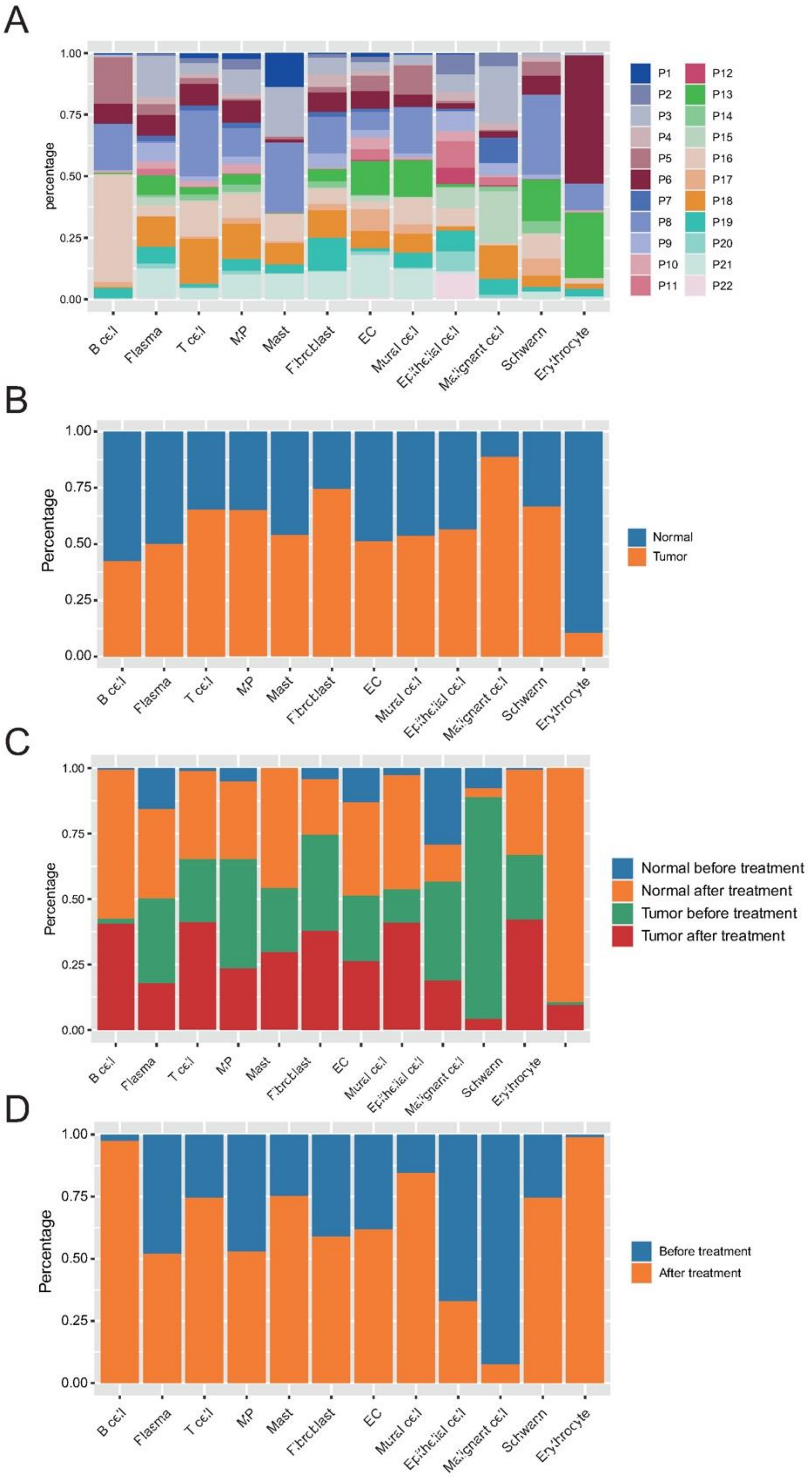


**Supplementary Figure 1.** (**A**) Bar chart showing the composition of samples within each cell population. (**B**) Bar chart showing the composition of tissue types within each cell population. (**C**) Bar chart showing the composition of tissue types within each cell population before and after

neoadjuvant therapy. (**D**) Bar chart showing the composition of neoadjuvant therapy status within each cell population.

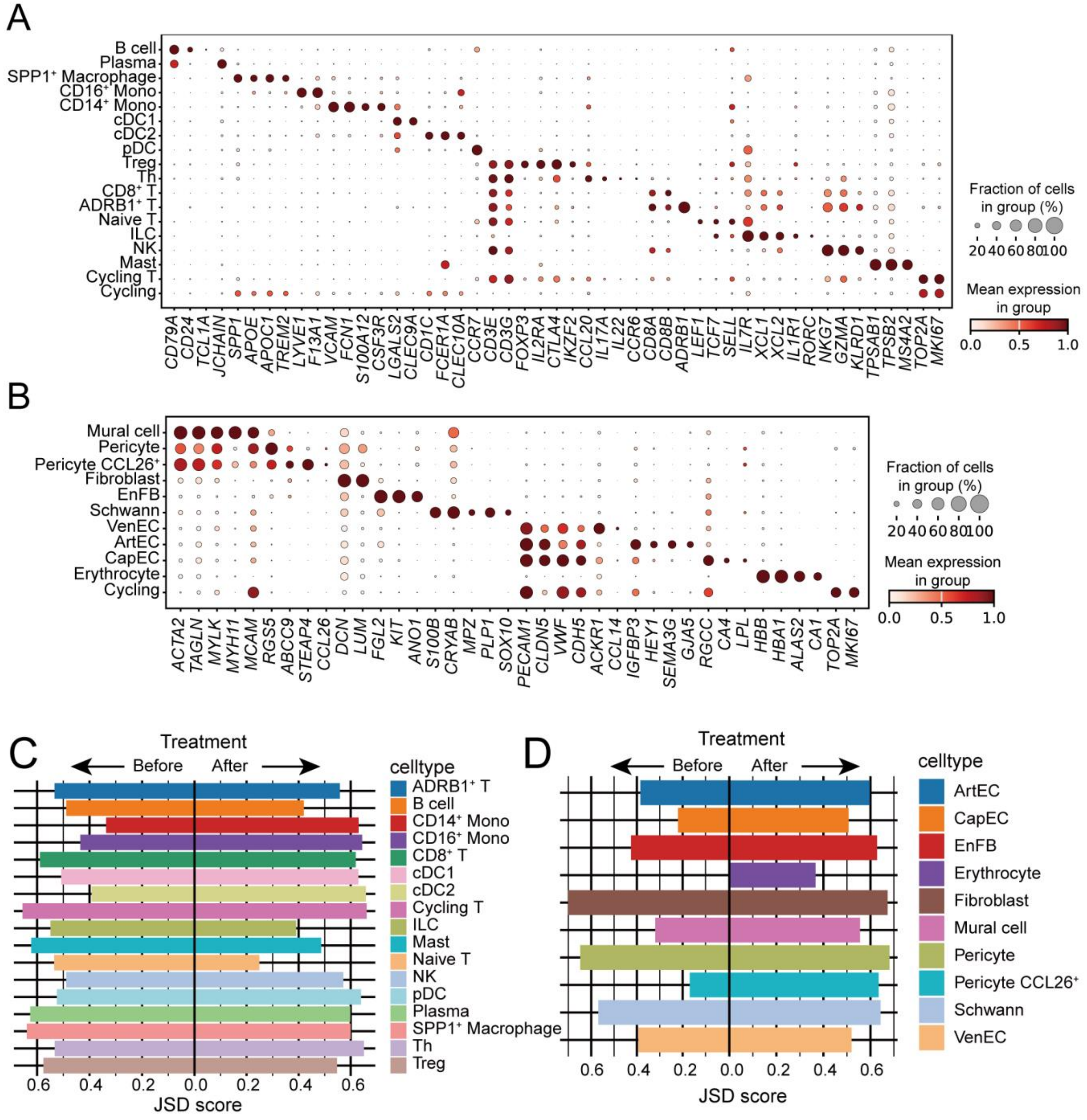


**Supplementary Figure 2.** (**A**) Dot plot showing marker genes for immune cell subtypes. The dot size represents the proportion of cells expressing a gene. Color denotes the scaled expression level. (**B**) Dot plot showing marker genes for stroma cell subtypes. The dot size represents the proportion of cells expressing a gene. Color denotes the scaled expression level. **(C)** Bar chart showing the Jensen–

Shannon divergence scores of each immune cell subtypes. **(D)** Bar chart showing the Jensen–Shannon divergence scores of each stroma cell subtypes.

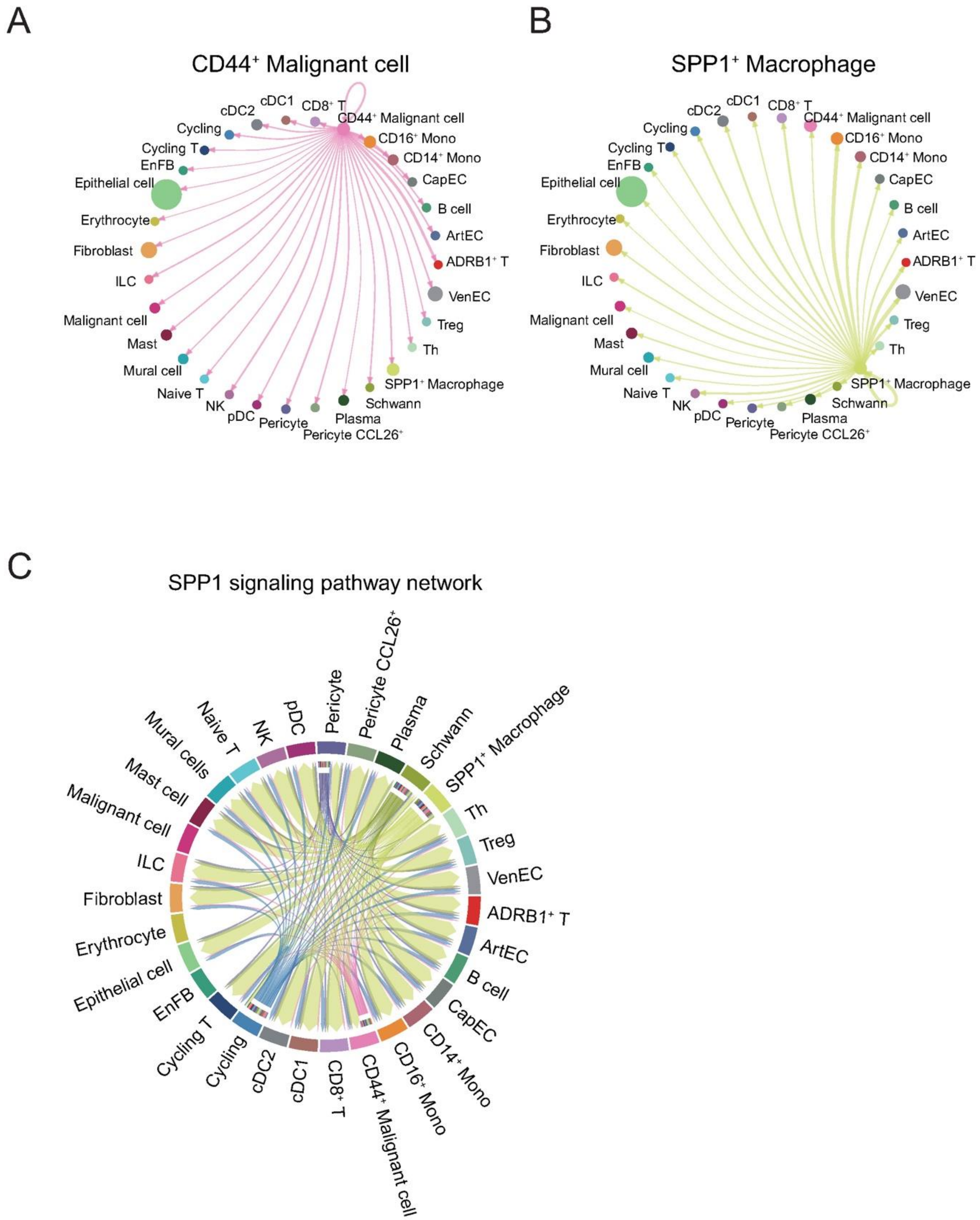

**Supplementary Figure 3.** (**A**) Screening of cellular interactions circle plots with CD44$^+$ Malignant cells as the source and tumor cells as the target. (**B**) Screening of cellular interactions circle plots with SPP1$^+$ macrophages as the source and tumor cells as the target. (**C**) Circle plot showing the strength of cellular interactions between SPP1$^+$ macrophages and other tumor cells on SPP1 signaling pathway.

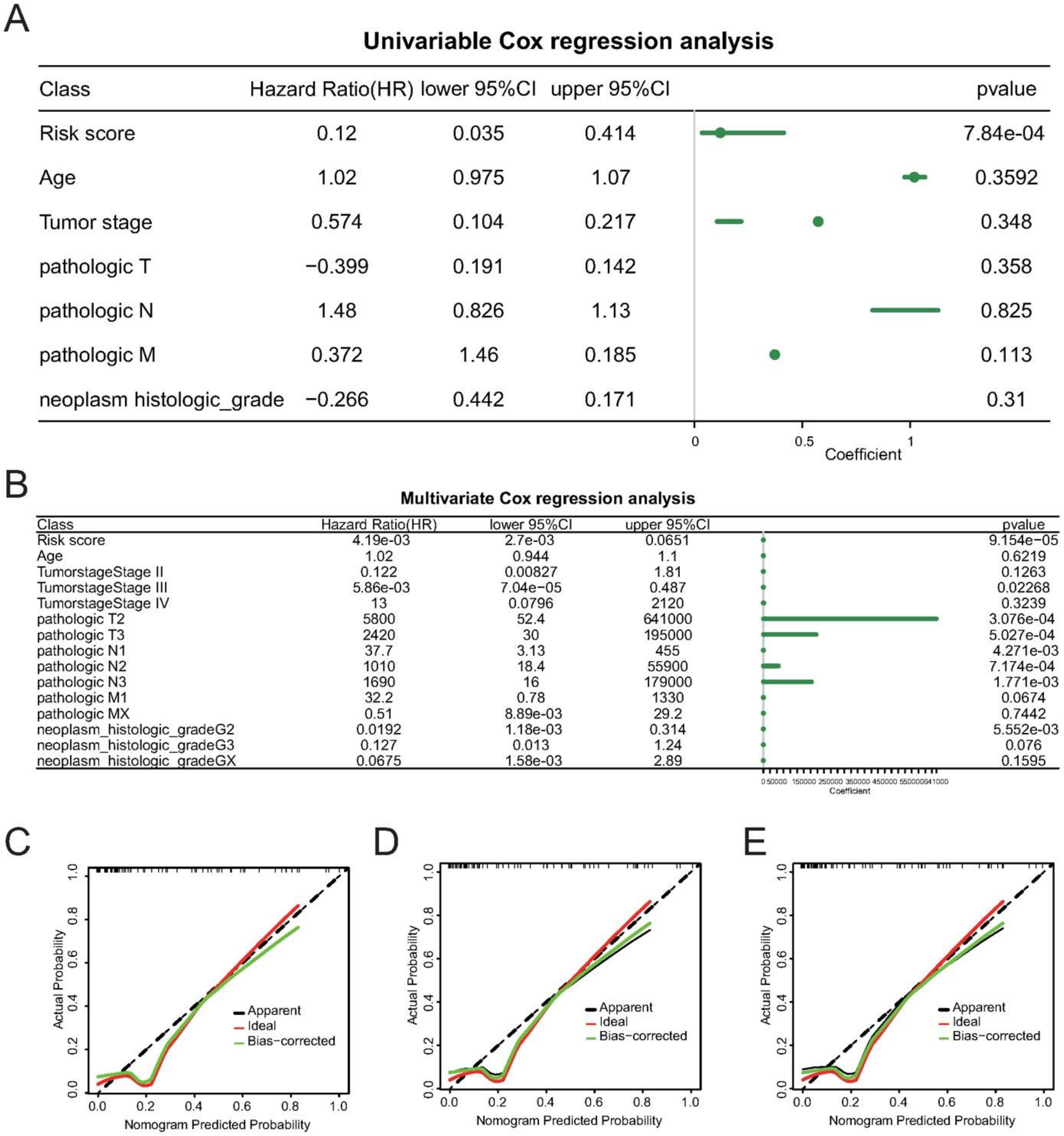


**Supplementary Figure 4.** (**A, B**) Univariate and multivariate Cox analyses evaluate the independent prognostic value of *ADRB1* risk signature in the training cohort. (**C-E**) Calibration graphs of the TCGA cohort's 1-year, 3-year and 5-year survival probabilities.
